# Chronic Infection by Plasmodium falciparum

**DOI:** 10.7759/cureus.53589

**Published:** 2024-02-04

**Authors:** José Pedro Manata, Marisa Brochado, Bernardo Silva, Jessenia Chinchila, João Matos Costa

**Affiliations:** 1 Internal Medicine, Hospital Distrital de Santarém, Santarém, PRT

**Keywords:** portugal, dna test, malaria, latent plasmodium falciparum, chronic infection

## Abstract

Malaria by Plasmodium falciparum (P. falciparum) usually does not exceed one year, but chronic infection, although rare, is a possibility. We present the clinical case of a 37-year-old male who came to the emergency department with intermittent fever, chills, and malaise. He had malaria more than 1 year ago while working in Huíla province, Angola. On admission, Plasmodium testing by light microscopy and antigens was negative. Doxycycline was started empirically, but on the third day of hospitalization, he had a new fever spike. Plasmodium DNA and antibodies were tested, confirming P. falciparum. The therapy with artemether-lumefantrine, already after discharge, allowed the consolidation of the treatment and eradicator of the parasite. Detection of parasite DNA by PCR should not be routine, but it is a more sensitive method, which confirmed this chronic infection by P. falciparum after one year.

## Introduction

Worldwide, in 2022, there were more than 249 million cases of malaria, and the estimated number of deaths was around 600,000. Africa accounts for more than 90% of malaria deaths. There are only imported cases in Portugal; the last endemic cases were diagnosed in 1959. Currently, there has been an increase in population migration between Portugal and the Portuguese-speaking African countries (PALOP), in which Angola is a good example [1-15].

Malaria is caused by a protozoan of the genus Plasmodium, which infects red blood cells [1,2]. The bite of the female Anopheles mosquito transmits it. Five different species of plasmodia are currently known to cause the disease in humans (Plasmodium falciparum (P. falciparum), Plasmodium vivax (P. vivax), Plasmodium malariae (P. malariae), Plasmodium ovale (P. ovale), and Plasmodium knowlesi (P. knowlesi)). The main plasmodium that transmits the disease in Angola is Plasmodium falciparum, considered the most malignant and responsible for the majority of cases [1-3].

Clinically, it presents as a general malaise, with high fever, chills, and profuse sweating, in cyclical patterns [1,2]. Initially, the symptoms are nonspecific, similar to a flu-like syndrome, but if treatment is delayed or ineffective, especially with P. falciparum infection, it can develop into severe malaria [1-5].

During the parasite's life cycle, the sporozoites are inoculated into the host and invade the hepatocytes. They mature into schizonts and rupture the liver cells, releasing the merozoites. Each merozoite invades an erythrocyte [6-11]. The initial symptoms of malaria, such as the typical fever and chills, are caused by the rupture of erythrocytes and their release of merozoites and other antigens, generating parasitemia [6-9].

The diagnosis of malaria is based on clinical suspicion and the detection of parasites in the blood. The two diagnostic methods routinely used are light microscopy and the detection of parasite-specific antigens or enzymes [6,12,13]. It is essential that treatment with antimalarials is administered as soon as possible and intravenously, consolidating treatment with an oral agent in order to eradicate the parasite and prevent recrudescence [3,5].

In P. vivax and P. ovale infections, schizonts in the liver can persist as hypnozoites for years, as dormant forms which, if not properly treated, can induce relapses and complicate treatment [2,3,5]. On the other hand, P. falciparum infections generally do not continue beyond one year, although there are records of cases in which it recurred one or two years after the primary infection [6,7]. Reinfection in endemic countries is a possibility, as are cases of transfusion malaria, in which the parasite continues its erythrocyte life cycle at very low densities [5,14].

## Case presentation

A 37-year-old male whose only relevant personal history is that he had malaria a year earlier when he was in Angola. He came to the emergency department with a fever, chills, and unwillingness going on for a week. The fever was intermittent, occurring every two days. Given the pandemic context, he decided to take the COVID test at the pharmacy, which came back negative.

On the initial physical examination, he had no fever and was hemodynamically stable. Analytically, he had leukopenia (leukocytes 3.8x10^9^/L) and thrombocytopenia (platelets 88,000x10^9^/L), a spontaneous International normalized ratio (INR) of 1.20 with a prothrombin time (PT) of 14.30 seconds and a prothrombin rate of 74.00%, but with a normal activated partial thrombin time (APTT), and an elevation of inflammatory parameters, C- reactive protein (CRP) of 13.29 mg/dL and procalcitonin of 3.31 ng/dL.

The Plasmodium antigen test and the thick drop blood smear were negative. SARS-COV-2 and HIV 1+2 tests were also negative. The urine test was normal, and the chest X-ray showed only slight bilateral hilar enhancement. 

It was decided to hospitalize him and start empirical Piperacillin/Tazobactam, later downgraded to Doxycycline. On the 3rd day of hospitalization, he had a fever again, of 38ºC, without chills, with normalization of the hematological picture and gradual reduction of CRP to 0.68 mg/dL. He underwent a body CT scan, which showed nothing relevant. On the 7th day of hospitalization, after completing the 5th day of Doxycycline and with no fever for more than three days, he was discharged.

Subsequent tests for P. falciparum, DNA, and total antibodies were positive. The high sensitivity of the PCR for P. falciparum raised additional concerns about eradicating this parasite, so a 3-day therapy with artemether-lumefantrine, 20 mg+120 mg, appropriate to the patient's weight, was implemented.

**Figure 1:**
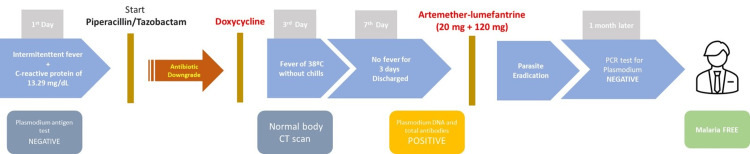
Time course of the clinical case.

One month later, the patient underwent a repeat PCR test for P. falciparum, the result of which was negative, thus confirming the diagnosis and reinforcing this therapeutic option. One month later, the patient underwent a repeat PCR test for P. falciparum, the result of which was negative, confirming the diagnosis and reinforcing this therapeutic decision (Figure 1).

## Discussion

Malaria caused by P. falciparum is characterized by a cyclical fever lasting 48/48 hours. If treatment is delayed or ineffective, it can develop into severe malaria, with a mortality rate of around 30% [2,3].

Initial treatment should be with intravenous quinine, the most widely used antimalarial, and a loading dose of 20 mg/kg of body weight should be administered, followed by a maintenance dose of 10 mg/kg every 8/8 hours. The reason for the loading dose is the urgent need to achieve therapeutic plasma concentrations, leading to a reduction in fever time and parasitemia, although no data shows its impact on mortality [2,3].

The current recommendations are at least 24 hours of parenteral therapy and, as soon as the patient tolerates oral therapy, supplementary treatment with an effective oral antimalarial such as Doxycycline (100 mg q12h) [2,3,14].

Primaquine remains the only schizonticide with a broad spectrum effective in the hepatic, asexual, and sexual erythrocyte stages to prevent relapse after infection, with the exception of P. falciparum [3]. This attitude is fundamental in P. vivax and P. ovale cases, where schizonts in the liver can persist for years.

In Angola, the most prevalent species is P. falciparum, but infections with this species generally do not last more than a year unless there is a reinfection or infection via transfusion [1,4]. The case of a 37-year-old male who had been working in Huíla province for a year, where he allegedly contracted malaria. There is no record of any transfusion. At the time, he was assisted and treated with antimalarials twice, as there was a recurrence after the first treatment.

More than a year later, he went to the ED with very similar symptoms. He was admitted for a fever of unclear etiology, with no signs of abscess or tumor on the CT scan. Given the strong clinical suspicion, it was decided to switch to Doxycycline in order to cover zoonoses, and also because of its antimalarial effect [2,3,14].

Routine parasite testing by light microscopy and antigen testing, carried out on admission and repeated during hospitalization, were always negative. Despite not being a routine method, the decision to confirm this infection by PCR revealed its high sensitivity and thus allowed the confirmation of chronic infection by P. falciparum after 1 year [2,3,6,13].

It is understood that the treatment with antimalarials, carried out in Angola, was incomplete, with the parasite maintaining its erythrocyte life cycle at low densities [13,14].

## Conclusions

Angola has already experienced a period of resistance to chloroquine, so it had to adopt a combination therapy based on Artemisinin to combat malaria. However, the worldwide spread of Plasmodium falciparum resistance to these combinations should be a concern. Given the possibility of severe malaria, early diagnosis of P. falciparum infection is essential.

Chronic infection with P. falciparum is rare, but it can happen. PCR is a more sensitive method for detecting the parasite's DNA, which has confirmed this chronic infection and allowed the parasite to be eradicated with artemether-lumefantrine. The mechanism by which the parasite remains undetected in the human host for long periods is still unclear, but it is known that incomplete treatment with antimalarials can have a modulating effect on the infection and even drug resistance.
